# Machine learning and weighted gene co-expression network analysis identify a three-gene signature to diagnose rheumatoid arthritis

**DOI:** 10.3389/fimmu.2024.1387311

**Published:** 2024-04-22

**Authors:** Ying-Kai Wu, Cai-De Liu, Chao Liu, Jun Wu, Zong-Gang Xie

**Affiliations:** ^1^ Department of Orthopaedic, The Second Affiliated Hospital of Soochow University, Jiangsu, China; ^2^ Department of Orthopaedics, Ningyang County First People’s Hospital, Tai an, China; ^3^ Department of General Practice, Affiliated Hospital of Weifang Medical University, Wei Fang, China; ^4^ Gynecology and Obstetrics, Ningyang County Maternal and Child Health Hospital, Tai an, China; ^5^ Medical Cosmetology and Plastic Surgery Center, LinYi People’s Hospital, Lin Yi, China

**Keywords:** rheumatoid arthritis, hub genes, machine learning, immune cell infiltration, WGCNA

## Abstract

**Background:**

Rheumatoid arthritis (RA) is a systemic immune-related disease characterized by synovial inflammation and destruction of joint cartilage. The pathogenesis of RA remains unclear, and diagnostic markers with high sensitivity and specificity are needed urgently. This study aims to identify potential biomarkers in the synovium for diagnosing RA and to investigate their association with immune infiltration.

**Methods:**

We downloaded four datasets containing 51 RA and 36 healthy synovium samples from the Gene Expression Omnibus database. Differentially expressed genes were identified using R. Then, various enrichment analyses were conducted. Subsequently, weighted gene co-expression network analysis (WGCNA), random forest (RF), support vector machine–recursive feature elimination (SVM-RFE), and least absolute shrinkage and selection operator (LASSO) were used to identify the hub genes for RA diagnosis. Receiver operating characteristic curves and nomogram models were used to validate the specificity and sensitivity of hub genes. Additionally, we analyzed the infiltration levels of 28 immune cells in the expression profile and their relationship with the hub genes using single-sample gene set enrichment analysis.

**Results:**

Three hub genes, namely, ribonucleotide reductase regulatory subunit M2 (*RRM2*), DLG-associated protein 5 (*DLGAP5*), and kinesin family member 11 (*KIF11*), were identified through WGCNA, LASSO, SVM-RFE, and RF algorithms. These hub genes correlated strongly with T cells, natural killer cells, and macrophage cells as indicated by immune cell infiltration analysis.

**Conclusion:**

*RRM2*, *DLGAP5*, and *KIF11* could serve as potential diagnostic indicators and treatment targets for RA. The infiltration of immune cells offers additional insights into the underlying mechanisms involved in the progression of RA.

## 1 Introduction

Rheumatoid arthritis (RA) is a systemic autoimmune disease characterized by chronic inflammation, proliferation of synovial membranes, and cartilage destruction, which has a serious impact on the physical and mental health of patients (1). Although RA does not directly lead to the mortality of patients, its systemic inflammatory damage can affect the function of organs such as the heart, lungs, and kidneys, reducing the quality of the patient’s life (2, 3). The pathogenesis of RA is complex and involves multiple factors such as genetics, environment, and metabolism. Moreover, the exact mechanisms associated with these factors and RA have not yet been systematically determined (4, 5). According to recent research, different types of immune cells, such as B cells, T cells, and macrophages, are closely associated with the development of RA (6). Other immune cells, including natural killer (NK) cells, mast cells, and dendritic cells (DCs) also play an important role in the development or advancement of RA (7–9).

Currently, studies on the treatment and pathogenesis of RA are increasing, but there is still a lack of highly specific and sensitive biomarkers for its early diagnosis. Bioinformatics is a discipline that combines biology, mathematics, and information technology and plays a prominent role in disease detection, biomarker identification, high-risk patient identification, and so on (10). Weighted gene co-expression network analysis (WCGNA) is a common method of identifying disease biomarkers and treatment targets. Machine learning algorithms, a subset of artificial intelligence that allows computers to learn from data and predict genes associated with disease, are also widely used in research (11). In our study, bioinformatics and three machine learning algorithms were comprehensively applied to integrate and analyze multiple expression datasets. This approach allowed the identification of highly sensitive and specific biomarkers and treatment targets and would provide new directions for subsequent experimental research. In this study, the expression matrix of four synovium samples was downloaded, the intersection genes were obtained by difference analysis and WGCNA, and then hub genes were identified by least absolute shrinkage and selection operator (LASSO), support vector machine–recursive feature elimination (SVM-RFE), and random forest (RF) machine learning algorithms, and their diagnostic efficiency was validated. Additionally, we analyzed the infiltration levels of 28 immune cells in the expression profile and their relationship with hub genes using single-sample gene set enrichment analysis (ssGSEA).

## 2 Materials and methods

### 2.1 Data collection and preprocessing

**Figure 1:**
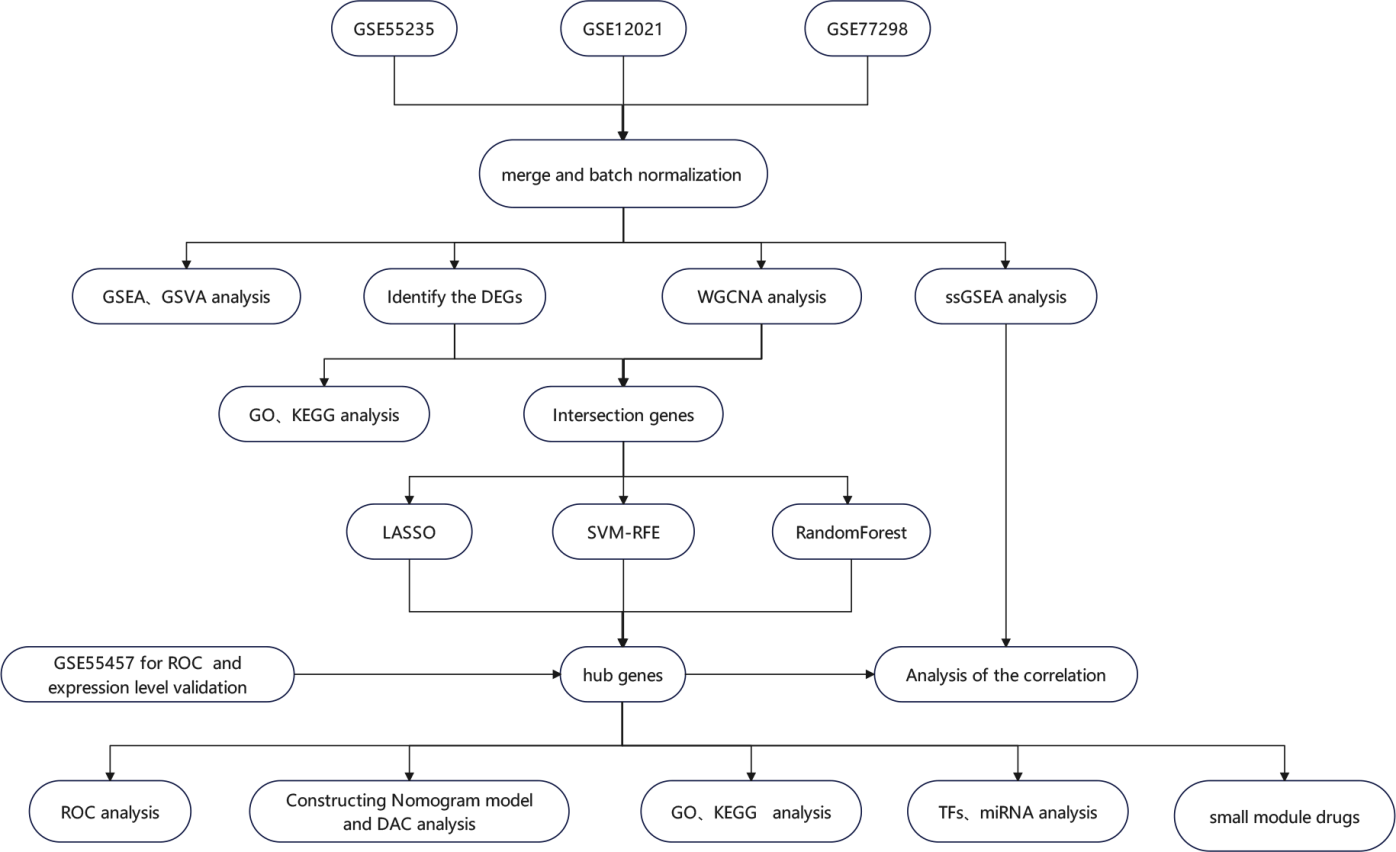
The flowchart depicting the investigation procedure. GSEA, gene set enrichment analysis; GSVA, gene set variation analysis; WGCNA, weighted gene co-expression network construction analysis; DEGs, differentially expressed genes; ssGSEA, single-sample gene set enrichment analysis; GO, Gene Ontology; KEGG, Kyoto Encyclopedia of Genes and Genomes; LASSO, least absolute shrinkage and selection operator; RF, random forest; SVM-RFE, support vector machine–recursive feature elimination; ROC, receiver operating characteristic curve; TFs, transcription factors; miRNAs, microRNAs; DCA, decision curve analysis.

The steps in the analysis of the entire research are shown in **Figure 1**. First, we obtained gene expression datasets of RA synovial samples (GSE77298, GSE55235, GSE12021, and GSE55457) from the Gene Expression Omnibus (GEO) database (https://www.ncbi.nlm.nih.gov/geo/) (12). These datasets included 87 synovial samples (36 normal control samples and 51 RA samples) (**Table 1**). GSE55457 was used as an external validation dataset, whereas the other datasets were merged and normalized for data analysis as a training set using the sva package (13). Common genes across each dataset were identified for further analysis.

**Table 1:** Information of datasets obtained from GEO.

Datasets	Platform	Total sample number	Normal sample number	RA sample number
GSE55235	GLP96	30	10	10
GSE77298	GLP96	23	7	16
GSE12021	GLP96	21	9	12
GSE55457	GLP570	33	10	13

### 2.2 Identification of differentially expressed genes and enrichment analyses

Differentially expressed genes (DEGs) were identified using the limma package with |log Fold Change (FC)| ≥ 1 and *P*-value < 0.05 used as the cutoff for filtering the DEGs (14). DEGs were visualized using a heatmap and volcano map obtained by using pheatmap and ggplot2 packages. Gene Ontology (GO) enrichment analysis and Kyoto Encyclopedia of Genes and Genomes (KEGG) analysis were conducted with a cutoff of *P* < 0.05 (15). A gene set variation analysis (GSVA) was performed using the GSVA R package to calculate a normalized enrichment score under the background of the hallmark gene set (c2.cp.kegg.v7.2) with the thresholds of the *P*-value and false discovery rate (FDR) set as 0.05 and 0.25, respectively (16). We also used GSEA to identify the biological attributes and functions of all genes in the training set by using clusterProfiler in the R package with significant thresholds selected as *P*-value < 0.05 and FDR < 0.25 (17).

### 2.3 Construction of the co-expression network

The WGCNA package was used to construct a weighted gene co-expression network (18). The samples were organized into clusters to identify outliers. Then, pairwise correlations were calculated between genes and a weighted adjacency matrix was constructed using a soft thresholding power β. The hierarchical clustering method was used to construct the clustering tree structure of the TOM(Topological overlap matrix). Different branches of the cluster tree represented different gene modules whcic were screened by different colours. To establish a link between modules and clinical characteristics, estimations of module membership (MM) and gene significance (GS) were computed. The modules with the highest Pearson coefficient and *P* < 0.05 were used to select the candidate hub genes under the criterion of MM > 0.8 and GS > 0.5.

### 2.4 Identifying hub genes

The Venn package was used to obtain intersecting DEGs and WGCNA candidate hub genes. LASSO logistic regression analysis was conducted using the R package glmnet with the optimal minimal lambda identified. Our study validated the selection of optimization parameters through 10-fold cross-validation, ensuring that the partial likelihood deviation satisfied the minimum criteria. The e1071 package was used to conduct the SVM-RFE with five-fold cross-validation, and the RF algorithm of the RF package was used to analyze the intersection genes. Ultimately, hub genes were obtained by identifying the overlapping genes derived from the three machine learning methods using a Venn diagram.

### 2.5 Constructing nomogram model and validation of hub genes

A nomogram for predicting RA was constructed using the rms package (19). The predictive power of the nomogram model was assessed using a calibration curve. A decision curve was used to assess the clinical utility of the nomogram model. A receiver operating characteristic (ROC) curve was created using the R package pROC function to determine the diagnostic value of the hub genes and the nomogram model for RA in the training and validation sets.

### 2.6 Correlation between immune cell infiltration and hub genes

The relative infiltration levels of 28 immune cells in the training set were quantified using the ssGSEA algorithm (20). Barplots were used to show the differential expression levels of 28 immune-infiltrating cells. Spearman correlations of 28 immune-infiltrating cells with hub genes were calculated and then visualized using the ggplot2 package.

### 2.7 Co-expression network of identified hub genes

GeneMANIA (https://genemania.org) was used to create a hub gene co-expression network (21).

### 2.8 Functional enrichment analysis of hub genes

The online tool Enrichr (22) (https://maayanlab.cloud/Enrichr/) was used to determine the biological process (BP), cellular component (CC), molecular function (MF), KEGG, WikiPathways, and Reactome enrichment analysis of the three hub genes (19). The significant threshold was adj. *P*-value < 0.05.

### 2.9 Transcription factors and microRNAs associated with the three hub genes

The JASPAR (https://jaspar.elixir.no/) database was used to find the transcription factors (TFs) that frequently bind to the three hub genes. MicroRNA (miRNAs) that interact with the hub genes were obtained from an online platform MirTarbase (https://mirtarbase.cuhk.edu.cn/).

### 2.10 Suppressive potential of small molecules in RA

We accessed the DSigDB (version 1.0) database through the Enrichr platform and obtained the top 10 small molecules that could suppress the expression of hub genes.

### 2.11 Statistical analysis

The statistical software R version 4.3.4 was used to perform statistical analysis, with *P*-values of < 0.05 indicating statistical significance.

## 3 Results

### 3.1 DEG identification

**Figure 2:**
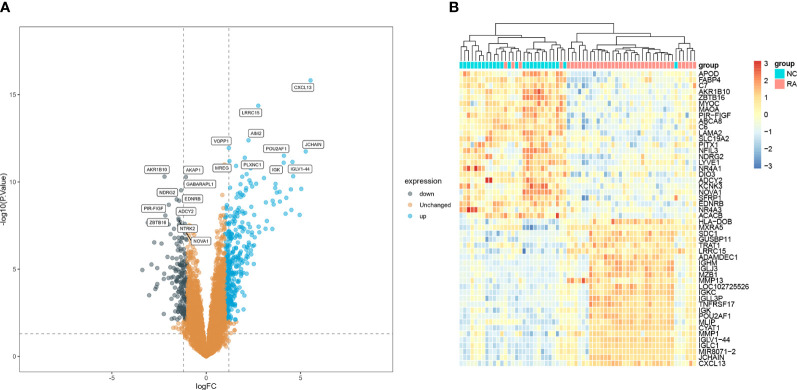
Identifications of RA hub genes. **(A)** Volcano plot and **(B)** heatmap present the identified DEGs between patients with RA and normal controls (|Iog FC| > 1 and adjusted p-value < 0.05 were defined as six screening standard to obtain DEGs). RA, rheumatoid arthritis; DEGs, differentially expressed genes.

A total of 575 DEGs (including 383 upregulated genes and 192 downregulated genes) were identified between RA and normal samples. The top 10 upregulated and downregulated DEGs are presented in a volcano plot (**Figure 2A**). In addition, the expression levels of the 25 most upregulated and 25 most downregulated genes are shown in a heatmap (**Figure 2B**).

### 3.2 Functional enrichment analysis

**Figure 3:**
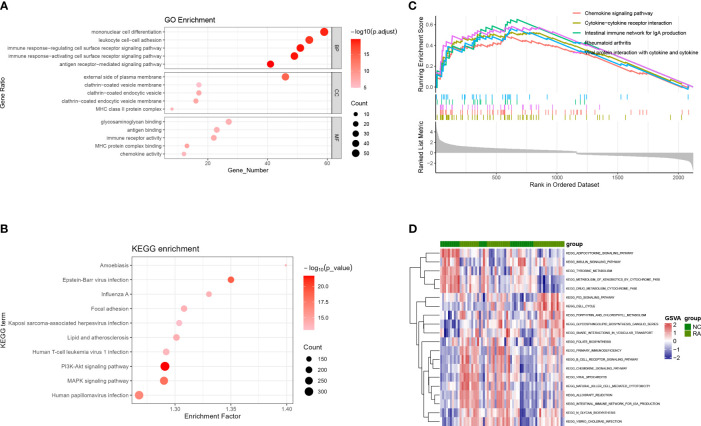
GO, KEGG, GSEA, and GSVA analyses based on GSE55235, GSE12021, and GSE77298. **(A)** Bubble diagram showing the GO enrichment analysis of DEGs. **(B)** Bubble diagram showing the KEGG enrichment analysis of DEGs. **(C)** GSEA analysis. **(D)** GSVA analysis. GO, Gene Ontology; KEGG, Kyoto Encyclopedia of Genes and Genomes; DEGs, differentially expressed genes; BP, biological process; MF, molecular function; CC, cellular component; GSEA, gene set enrichment analysis; GSVA, gene set variation analysis.

DEGs in the BP category of GO were enriched in mononuclear cell differentiation, leukocyte cell–cell, and immune response–regulating cell surface receptor signaling pathways. In the MF category, DEGs were mostly related to antigen binding, immune receptor activity, and chemokine activity. In the CC category, DEGs were mostly assigned to the external side of the plasma membrane and clathrin-coated vesicle membrane (**Figure 3A**). KEGG pathway analysis showed that DEGs were enriched in cytokine–cytokine receptor interaction, chemokine signaling pathway, and RA; GSEA analysis produced similar results (**Figure 3B**). GSEA was used to depict the signal pathways involved in RA. The top five pathways enriched by DEGs were the chemokine signaling, cytokine–cytokine receptor interaction, intestinal immune network for Immunoglobulin A (IgA) production, RA, viral protein interactions with cytokines, and cytokine (**Figure 3C**). In the RA group, the GSVA results of enriched DEGs also indicated that immunity and inflammation pathways, such as chemokine signaling, NK cell–mediated immunity, B-cell receptor signaling, primary immunodeficiency, and intestinal immune network for IgA production were evident (**Figure 3D**).

### 3.3 WGCNA construction and hub module identification

**Figure 4:**
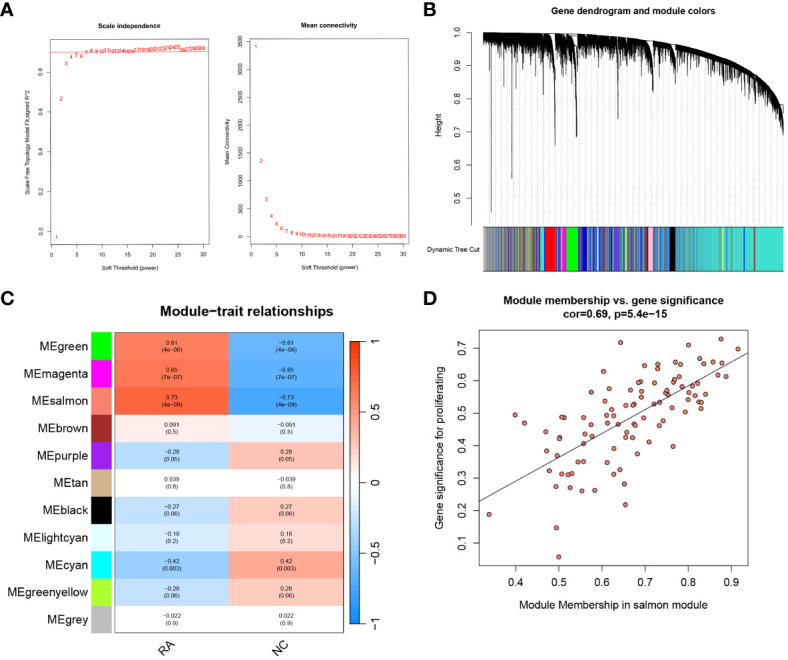
WGCNA analysis and hub candidates for RA. **(A)** Analysis of the mean connectivity and scale-free fit index for different soft-thresholding powers (β). Where the correlation coefficient is 0.9 and the matching soft-thresholding power is 8, the red line represents this location. **(B)** The cluster dendrogram of the top 25% of genes median absolute deviations. Each hue in the graphic below corresponds to a co-expression module, and each branch in the figure represents a single gene. **(C)** Heatmap illustrating the relationships between modules and traits. The salmon module has a strong correlation with RA. **(D)** Scatter plot showing the relationship between the genes relevance and its inclusion in the salmon module of genes. WGCNA, weighted gene co-expression network analysis.

Samples in the training set were clustered using the WGCNA package. Subsequently, the unscaled connectivity index was determined, and an average connectivity analysis was conducted. When the soft threshold β = 8, the network reached an unscaled topological threshold of 0.9 (**Figure 4A**). By dynamic tree cutting and calculation, 11 gene modules were obtained (**Figure 4B**). Correlation analysis was performed between the 11 modules and the normal and RA groups, resulting in a correlation heatmap (**Figure 4C**). The salmon module had the strongest correlation with the RA (r = 0.73, *P* < 0.001) and was identified as the key module for RA. Based on filtering criteria, we identified 17 candidate hub genes in the salmon module (**Figure 4D**).

### 3.4 Screening of hub genes

**Figure 5:**
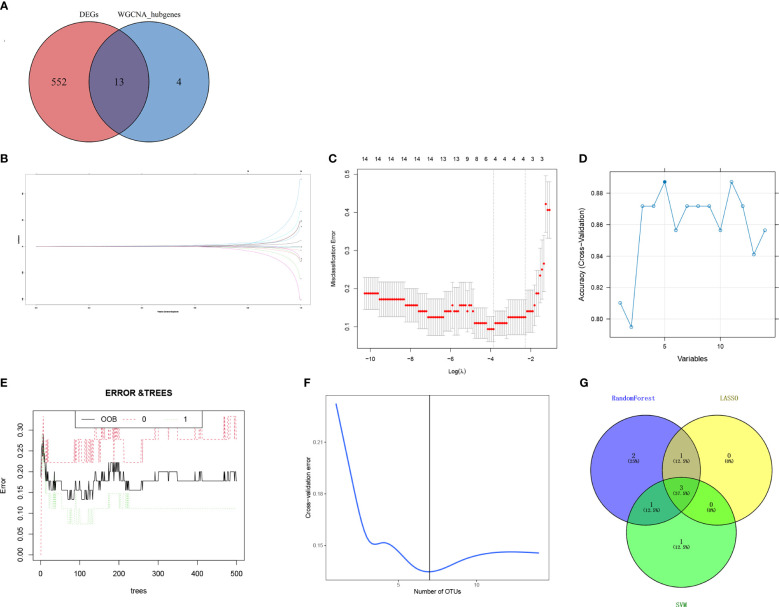
Screening of hub genes. **(A)** Venn diagram for overlapped genes between DEGs and WGCNA. **(B)** The LASSO regression partial likelihood deviance with changing log (λ) plotted in 10-fold cross-validations. Dotted vertical lines were drawn at the optimal values using the minimum criteria (lambda.min) and 1 standard error of the minimum criteria (1-SE criteria). **(C)** The LASSO coefficient profiles for four hub genes in the 10-fold cross-validation. The intersection of **(D)** five gene signatures was identified by SVM-RFE analysis. **(E)** Prediction accuracy of the RandomForest model. **(F)** Seven gene signatures were identified by RandomForest analysis. **(G)** Overlapped genes obtained from the LASSO, SVM-RFE, and random forest algorithms. DEGs, differentially expressed genes; WGCNA, weighted gene co-expression network analysis; LASSO, least absolute shrinkage and selection operator; SVM-REF, support vector machine–recursive feature elimination.

By intersecting the DEGs and candidate hub genes, 13 intersection genes were obtained (**Figure 5A**). The 13 intersection genes were then submitted into three machine learning algorithms including LASSO, SVM-RFE, and RF. LASSO resulted in four hub genes (**Figures 5B, C**), SVM identified five hub genes (**Figure 5D**), and RF identified seven hub genes (**Figures 5E, F**). Finally, we obtained three hub genes ribonucleotide reductase regulatory subunit M2 (*RRM2*), DLG-associated protein 5 (*DLGAP5*), and kinesin family member 11 (*KIF11*) by intersecting the three machine learning results (**Figure 5G**).

### 3.5 Constructing the nomogram model and validation

**Figure 6:**
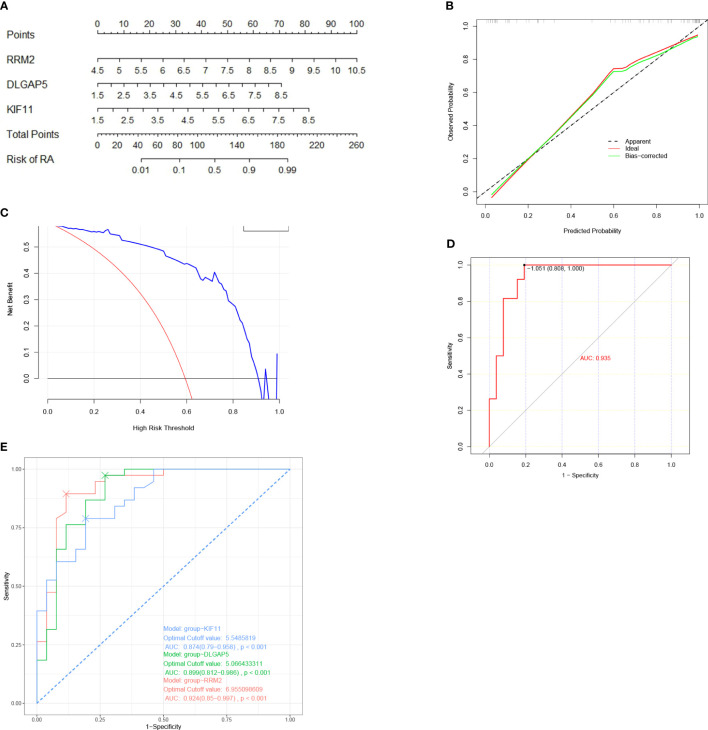
Nomogram model construction for RA diagnosis. **(A)** Nomogram to predict RA risk. **(B)** Calibration curve evaluation for the diagnostic potential of the nomogram model. **(C)** DCA curve to assess the nomogram practical efficacy. **(D)** ROC analysis of the model. **(E)** ROC analysis of three hub genes. DCA, decision curve analysis; ROC, receiver operating characteristic; AUC, area under the ROC curve (based on GSE77298, GSE55235, and GSE12021).

**Figure 7:**
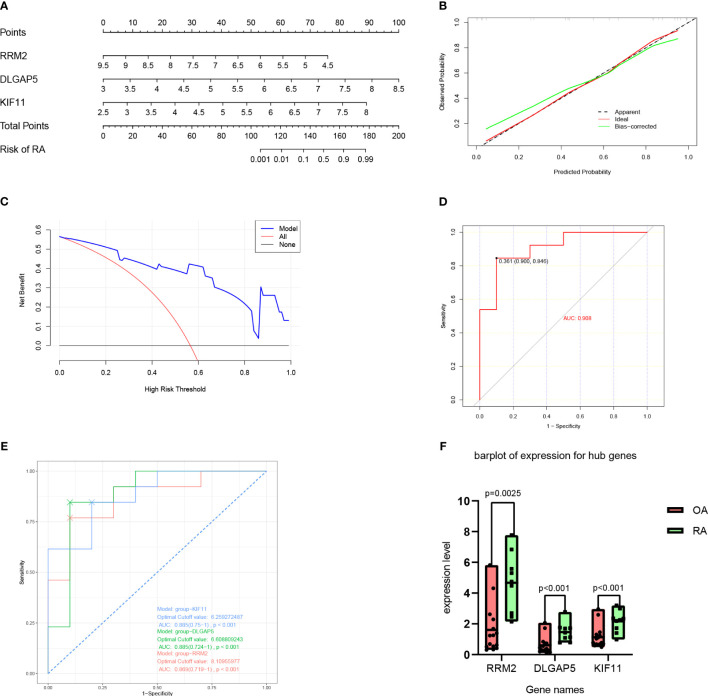
Nomogram model construction for RA diagnosis. **(A)** Nomogram to predict RA risk. **(B)** Calibration curve evaluation for the diagnostic potential of the nomogram model. **(C)** DCA curve to assess the nomogram practical efficacy. **(D)** ROC analysis of the model. **(E)** ROC analysis of three hub genes. **(F)** Expression level of hub genes according to datasets from GitHub by Shanghai Guanghua Hospital. DCA, decision curve analysis; ROC, receiver operating characteristic; AUC, area under the ROC curve (based on GSE55457).

A nomogram model was then constructed using the three hub genes in the training set to predict the risk of RA (**Figure 6A**). The nomogram model was found to have the best predictive and clinical efficiency for RA by calibration curves (**Figure 6B**) and decision curve analysis (**Figure 6C**), respectively. The area under the ROC curve (AUC) of the nomogram model and three hub genes were also calculated (**Figures 6D, E**). Next, we constructed a validation set using all the procedures, which showed a perfect match with the resultsin the training set. To further validate this result, we obtained RNA-seq data on the synovium of patients with RA and osteoarthritis (OA) uploaded to GitHub by Shanghai Guanghua Hospital (23) and analyzed the expression levels of key genes in the data. Consistent with this study, we found that the expression levels of three hub genes in RA synovium were significantly higher than in OA synovium (*P* < 0.05) (**Figure 7**).

### 3.6 Correlation between the immune cell infiltration and hub genes

**Figure 8:**
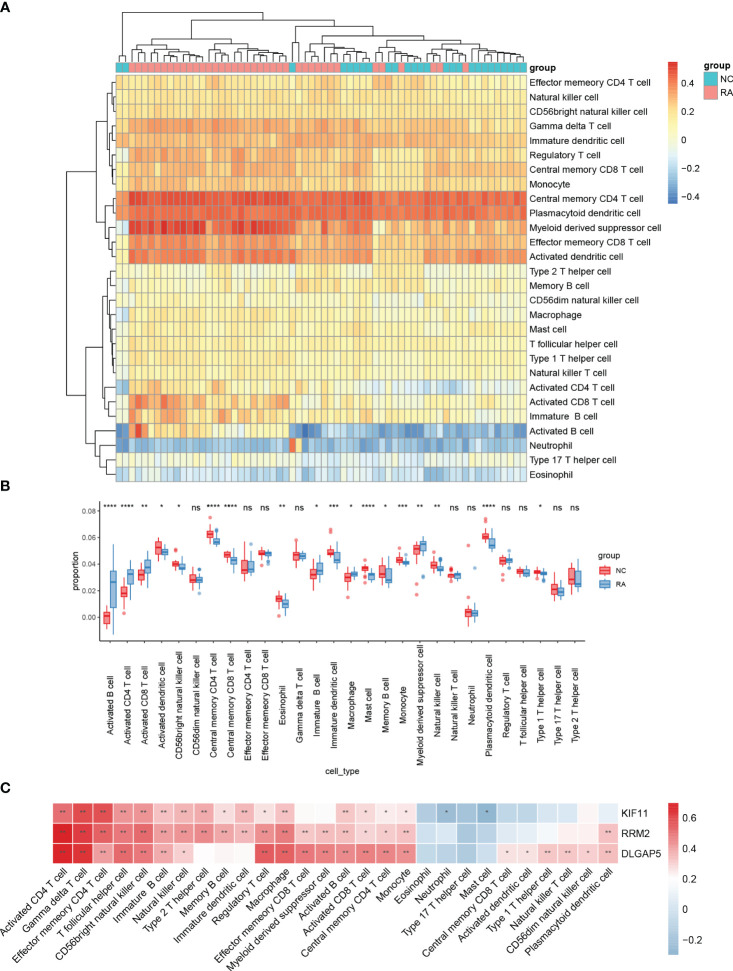
Analyses of the OA-related immunological environment. The distribution of 28 different types of immune cells in healthy control and OA synovial tissues is shown in a heatmap **(A)** and a violin plot **(B)**. **(C)** The association between immune cells infiltration and four hub genes. ****, ***, **, * represented P<0.0001, P<0.001, P<0.01, P<0.05.

The distribution of 28 immune cells in the training set is demonstrated in **Figure 8A**. In our results, a significantly higher infiltration of activated CD4 T cells, activated B cells, and activated DC infiltration was found in RA, indicating the important role that they play in the disease (**Figure 8B**). Correlation analysis of the 28 immune cells with hub genes demonstrated that various T cells, B cells, NK cells, and macrophages were positively correlated with the three hub genes (**Figure 8C**).

### 3.7 Function analysis of hub genes

**Figure 9:**
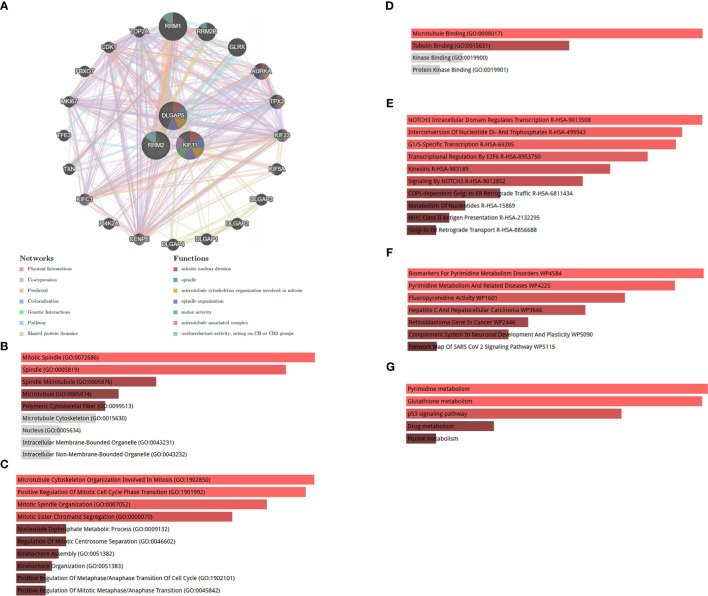
**(A)** Co-expression network of hub genes. Hub genes and their co-expression genes were analyzed via GeneMANIA. **(B)** Significantly enriched cellular components. **(C)** Significantly enriched biological processes. **(D)** Significantly enriched molecular functions. GO, Gene Ontology. **(E)** Reactome pathway, **(F)** WikiPathway, and **(G)** KEGG 2021 human pathway. KEGG, Kyoto Encyclopedia of Genes and Genomes.

To analyze the biological functions of the identified hub genes, we constructed a comprehensive gene interaction network using data from the gene MANIA database (**Figure 9A**). This network comprised physical interactions, co-expression relationships, predicted interactions, co-localization patterns, genetic interactions, pathway interactions, and shared protein domains. Our findings indicate that the hub genes are primarily associated with mitotic nuclear division, spindle, and microtubule cytoskeleton organization involved in mitosis and spindle organization. Furthermore, to discern the specific biological roles of the three hub genes, we conducted an enrichment analysis. In **Figures 9B–D**, we illustrate the most enriched terms in the CC, BP, and MF analyses of GO terms. Additionally, **Figures 9E–G** depict the most significant pathways based on data from the Reactome, Wiki Pathway, and KEGG databases, respectively.

### 3.8 Identification of regulatory signatures

**Figure 10:**
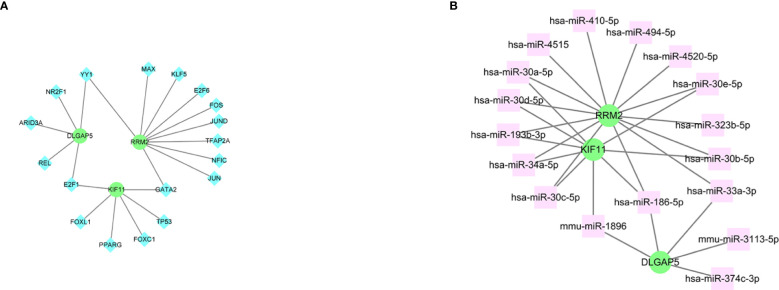
The cohesive regulatory interaction network of three hub genes and TFs and miRNAs obtained from the Network Analyst. **(A)** Genes and TFs. **(B)** Genes and miRNAs. Herein, the diamond nodes are TFs; the square node indicates miRNAs; gene symbols as circle nodes. TF, transcription factors; miRNAs, microRNAs.

The interplay between the three hub genes and TF regulators is depicted in **Figure 10A**, whereas the relationships between the hub genes and miRNA regulators are illustrated in **Figure 10B**. In total, we identified 18 TFs and 17 miRNAs as regulatory signatures by analyzing TF–gene and miRNA–gene interaction networks.

### 3.9 Discovery of potential small molecules

We generated potential small-molecule findings based on odds ratios. **Table 2** presents the top 10 small molecules that could potentially target the hub genes sourced from the DSigDB database.

**Table 2:** Top 10 small-molecule drugs for RA.

Term	Overlap	P-value	Adjusted P-value	Odds ratio	Combined score	Genes
LUCANTHONE CTD 00006227	3/213	1.19E-06	1.86E-04	59361	809723.5033	RRM2;KIF11;DLGAP5
0173570-0000 PC3 DOWN	2/43	1.35E-05	5.35E-04	973.4634146	10913.3726	KIF11;DLGAP5
Phytoestrogens CTD 00007437	2/48	1.69E-05	5.35E-04	867.4347826	9531.856143	RRM2;DLGAP5
Etoposide MCF7 DOWN	2/48	1.69E-05	5.35E-04	867.4347826	9531.856143	KIF11;DLGAP5
Methotrexate MCF7 DOWN	2/52	1.99E-05	5.35E-04	797.88	8638.620968	KIF11;DLGAP5
Piroxicam CTD 00006571	3/549	2.06E-05	5.35E-04	58353	629719.0634	RRM2;KIF11;DLGAP5
Troglitazone CTD 00002415	3/651	3.43E-05	7.65E-04	58047	596691.1364	RRM2;KIF11;DLGAP5
Apigenin MCF7 DOWN	2/87	5.60E-05	0.0010851	468.5176471	4587.228583	RRM2;KIF11
Pyrvinium MCF7 DOWN	2/92	6.26E-05	0.0010851	442.3777778	4281.648144	RRM2;KIF11
Resveratrol MCF7 DOWN	2/104	8.01E-05	0.001249052	390.0980392	3679.655006	KIF11;DLGAP5

## 4 Discussion

RA is a chronic inflammatory disease that currently lacks early diagnostic indicators (6). Recent studies have highlighted the close association of various immune cells, such as B cells, T cells, and macrophages, with the pathogenesis of RA (24). Therefore, the exploration of new diagnostic biomarkers and their relationship with immune cell infiltration patterns holds significant implications for advancing our understanding of RA pathophysiology. To address this, we gathered four RA synovial microarray datasets from the GEO database and identified 575 DEGs between RA and healthy controls. Enrichment analyses, GO, KEGG, GSEA, and GSVA revealed a robust correlation between RA and the immune response.

Fibroblast-like synoviocytes (FLSs) are the most abundant cells of the stroma and a key population in RA. Recent research indicates that the interaction between RA FLSs and infiltrating immune cells is pivotal in chronic inflammation and bone degradation. Specifically, CD4+ T helper cells, T helper cell 1 (Th1) and T helper cell 17 (Th17) cells, produce cytokines that either inhibit or stimulate osteoclast formation, impacting bone health. The involvement of T cells in bone loss was first demonstrated in 1999, highlighting their role in promoting osteoclastogenesis and subsequent bone erosion. Studies using mouse models further support the significance of Th17 cells and Interleukin-17 (IL-17) in bone damage, with therapeutic interventions targeting IL-17 showing promise but not leading to complete disease remission in patients with RA (25).

Our findings revealed that macrophages play an important role in the infiltration of immune cells in the synovium. In contrast to tissue-resident macrophages, infiltrating macrophages may originate from various monocyte subpopulations in the blood and possess a high level of adaptability. For instance, in mice, they can arise from classical Ly6C+ or patrolling Ly6C^−^ monocytes (26, 27). In a recent comprehensive analysis of immune cell status in patients with RA, single-cell RNA-seq, bulk RNA-seq, and mass spectrometry flow cytometry were used to identify 18 distinct synoviocyte populations, including four monocyte/macrophage populations denoted as SC-M1 to SC-M4 (28). This analysis demonstrated that the activation of different cytokines promoted the expansion of diverse macrophage subpopulations in the RA synovium. Furthermore, as the primary orchestrators of the immune response, DCs can secrete chemokines that facilitate the activation of inflammatory T cells, thereby attracting proinflammatory immune cells such as macrophages and neutrophils (29–31). *In vitro*, RA synovial DCs have the potential to induce regulatory T (Treg) cells through the prolonged engagement of programmed cell death 1 receptors (32, 33). Although Treg cells in the peripheral blood of patients with RA retain inhibitory capacity, this function is compromised in local Treg cells, suggesting that the inflammatory cytokine environment may contribute to Treg cell dysfunction (34).

Through the use of WGCNA and machine learning algorithms, we identified *DLGAP5*, *RRM2*, and *KIF11* as potential diagnostic markers for RA. *RRM2* plays a critical role in controlling the production of deoxyribonucleotides, which is essential for DNA repair and synthesis (35). Blocking *RRM2* has a substantial impact on reducing cellular growth and triggering cell death (36, 37). Recently, other studies have demonstrated that *RRM2* could increase the levels of apoptosis and inhibit the proliferation of RA-FLSs by regulating transforming growth factor-β (TGF-β) and IL-6 (38).

Although several bioinformatics methodologies have been used to investigate potential biomarkers for RA, there is limited literature regarding the involvement of *DLGAP5* in the pathophysiology of this condition (39, 40). Previous investigations have examined the structure and function of *DLGAP5* across various species, considering both physiological and clinicopathological perspectives. These studies have revealed that *DLGAP5* plays a crucial role in facilitating cell growth, proliferation, and migration (41, 42). Therefore, this presents an opportunity to investigate in further detail the potential of *DLGAP5* in diagnosing and differentially diagnosing RA, as well as its role in the pathophysiology of the disease.

*KIF11* encodes a motor protein belonging to the kinesin-like protein family, which is recognized for its involvement in diverse spindle dynamics. The role of the gene product encompasses chromosome positioning, centrosome separation, and the establishment of a bipolar spindle during cell mitosis (43). However, there is limited literature on the role of KIF11 in the RA joint microenvironment. Therefore, in this study, *KIF11* along with the two other hub genes performed a diagnosis of RA with optimal sensitivity and specificity.

This study has several limitations. First, the dataset obtained from the GEO database lacks comprehensive patient information, including serological and imaging indicators. As a result, we were unable to evaluate the correlation of biomarkers or immune cells with clinical characteristics such as hematological indicators, degree of joint destruction, and treatment status in patients with RA. More detailed data are necessary for the further exploration of the clinical significance of the biomarkers. Second, the biomarker discovery was based on the GEO database. Despite the satisfactory performance of our biomarkers in both test and validation datasets, additional *in vitro* and *in vivo* experiments are required to validate our findings and determine the mechanisms underlying significant immunological changes during RA.

## 5 Conclusion

Using LASSO, SVM-RFE, and RF algorithms in conjunction with bioinformatic analyses, we identified a three-gene signature (*RRM2, DLGAP5*, and *KIF11*) implicated in the progression of RA. Immune infiltration analyses revealed that the identified hub genes exhibited the strongest correlation with various T cells, B cells, NK cells, and macrophages. To confirm our identification of diagnostic markers with high sensitivity and specificity for RA, prospective large-sample investigations with experimental validation should be conducted.

## Data availability statement

The original contributions presented in the study are included in the article/supplementary material. Further inquiries can be directed to the corresponding author.

## Ethics statement

Ethical approval was not required for the study involving humans in accordance with the local legislation and institutional requirements. Written informed consent to participate in this study was not required from the participants or the participants’ legal guardians/next of kin in accordance with the national legislation and the institutional requirements. The manuscript presents research on animals that do not require ethical approval for their study.

## Author contributions

Y-KW: Writing – original draft. C-DL: Data curation, Writing – review & editing. CL: Formal analysis, Writing – review & editing. JW: Formal analysis, Writing – review & editing. Z-GX: Supervision, Validation, Writing – review & editing.
